# Distinct Fiber Type Signature in Mouse Muscles Expressing a Mutant Lamin A Responsible for Congenital Muscular Dystrophy in a Patient

**DOI:** 10.3390/cells6020010

**Published:** 2017-04-24

**Authors:** Alice Barateau, Nathalie Vadrot, Onnik Agbulut, Patrick Vicart, Sabrina Batonnet-Pichon, Brigitte Buendia

**Affiliations:** 1 Unité de Biologie Fonctionnelle et Adaptative (BFA), CNRS UMR 8251, Université Paris Diderot, Sorbonne Paris Cité, 75013 Paris, France; nathalie.vadrot@univ-paris-diderot.fr (N.V.); patrick.vicart@univ-paris-diderot.fr (P.V.); sabrina.pichon@univ-paris-diderot.fr (S.B.-P.); brigitte.buendia@univ-paris-diderot.fr (B.B.); 2 Biological Adaptation and Ageing, UMR CNRS 8256, Institut de Biologie Paris-Seine (IBPS), UPMC Univ Paris 06, Sorbonne Universités, 75005 Paris, France; onnik.agbulut@upmc.fr

**Keywords:** lamin A, congenital muscular dystrophy, muscle fiber type transition, myosin heavy chain IIA

## Abstract

Specific mutations in *LMNA*, which encodes nuclear intermediate filament proteins lamins A/C, affect skeletal muscle tissues. Early-onset *LMNA* myopathies reveal different alterations of muscle fibers, including fiber type disproportion or prominent dystrophic and/or inflammatory changes. Recently, we identified the p.R388P *LMNA* mutation as responsible for congenital muscular dystrophy (L-CMD) and lipodystrophy. Here, we asked whether viral-mediated expression of mutant lamin A in murine skeletal muscles would be a pertinent model to reveal specific muscle alterations. We found that the total amount and size of muscle fibers as well as the extent of either inflammation or muscle regeneration were similar to wildtype or mutant lamin A. In contrast, the amount of fast oxidative muscle fibers containing myosin heavy chain IIA was lower upon expression of mutant lamin A, in correlation with lower expression of genes encoding transcription factors MEF2C and MyoD. These data validate this in vivo model for highlighting distinct muscle phenotypes associated with different lamin contexts. Additionally, the data suggest that alteration of muscle fiber type identity may contribute to the mechanisms underlying physiopathology of L-CMD related to R388P mutant lamin A.

## 1. Introduction

A-type lamins (A and C) are nuclear intermediate filament proteins. In mammalian cells, these proteins play multiple roles due to their assembly into polymers beneath the nuclear envelope as well as in the nucleoplasm and their capacity to bind multiple partners and chromatin [1,2]. Thus, A-type lamins regulate the shape and mechanical properties of nuclei and expression of genes related to cell proliferation and differentiation processes. Mutations in *LMNA*, which encodes lamins A and C, cause several diseases that eventually affect cardiac and/or skeletal muscle tissues [3]. Some mouse models have been developed to address the impact of mutant lamin proteins on different organs in vivo [4]. We previously showed that adeno-associated virus (AAV) expressing ectopic proteins can be directly injected into mouse skeletal muscles [5], providing a useful approach for assessing protein impact in vivo. 

The fast-twitch muscle tibialis anterior (TA) is poor in type I fibers expressing myosin heavy chain 1 (MHC I), which have oxidative metabolism, but rich in type II fibers expressing MHC II isoforms [6]. Among the latter, one can distinguish type IIA fibers (MHC IIA) with an oxidative metabolism, also named intermediate fibers, and type IIX and IIB fibers (MHC IIX and IIB) with a glycolytic metabolism. Of note, in humans, the Myh4 gene is present but the protein MHC IIB is not expressed, In addition, some fibers can express different myosin heavy chain isoforms resulting in hybrid fibers with various properties in terms of ATP usage and muscle contraction speeds [7]. Muscle fibers with an oxidative metabolism are more resistant to fatigue than those with a glycolytic metabolism. Of note, the proportion of muscle fiber types varies depending on muscle specificity and activity [7]. For instance, physical training to increase endurance favors a shift towards more fatigue-resistant MHC I and MHC IIA type fibers. 

In addition, physiopathological contexts can induce fiber type disproportion (FTD). In particular, congenital myopathies triggered by some *LMNA* mutations (p.Glu33del, p.Lys123del, p.Ser303Pro, p.Arg453Trp, p.Thr528Lys) can induce a particular form of muscle FTD characterized by type II fiber hypertrophy [8]. Similar FTD is also observed in other *LMNA* related myopathies such as limb-girdle muscle dystrophy (*LMNA* mutation p.T27I) [9]. Another type of FTD associated with congenital myopathies caused by mutations in other genes, including *ACTA1*, *MYH7*, or *TMP2,3*, is specified by an abnormally high amount of slow oxidative type I fibers that are also atrophic [8,9,10].

We recently identified one particular lamin A mutant, *LMNA* p.R388P, that is responsible for congenital muscular dystrophy (L-CMD) [11]. Here, we used AAV injections to rapidly assess the impact of R388P mutant lamin A on mouse skeletal muscle homeostasis. Our analysis included morphological features of TA fibers; relative oxidative potential of muscle fibers, using quantification of succinate dehydrogenase (SDH) enzyme; and fiber type identity based on content of different MHC isoforms.

## 2. Materials and Methods 

### 2.1. Plasmid Production

Plasmids encoding FLAG-tagged human WT and mutant lamin A under regulation of the CMV promoter [11] were used by the Therapeutic Research Institute—Institut de Recherche en Santé de l’Université de Nantes (IRS-UN), Centre de Production de Vecteurs viraux (CPV), Institut National de la santé et de la recherche médicale (INSERM), Unité Mixte de Recherche (UMR) 1089 (Nantes, France)— to generate and purify AAV2/2.CMV.FLAG.LA WT or R388P. Final viral preparations were stored in Dulbecco’s Phosphate-Buffered Saline (DPBS) (with Ca^2+^ and Mg^2+^), aliquoted in small volumes (50 μL), and stored at −80 °C. Particle titer (number of viral genomes) was determined by quantitative PCR poly A.

### 2.2. Intramuscular Delivery of AAV Vectors 

All animal protocols were approved by the institutional ethics committee and conducted according to French and European laws, directives, and regulations on animal care (European Commission Directive 86/609/European Economic Community (EEC); project number #5441 at the Comité d'éthique en expérimentation animale Charles Darwin N°5). The Centre d’expérimentation Fonctionnelle (CEF) animal facility at Hopital Salpétrière in Paris is fully licensed by the competent French authorities and has animal welfare insurance. For intramuscular injections, 10-week-old female C57BL/6J mice (Janvier Labs, Le Genest-Saint-Isle, France) were randomized into two groups: AAV-FLAG-LA-WT and AAV-FLAG-LA-R388P. Animals were anesthetized by intraperitoneal injection of a mix of drugs with sedative and analgesic effects [10 mg/kg xylazine (Bayer, Puteaux, France) and 100 mg/kg ketamine (Merial, Gerland, France)]. Insulin syringes were used to inject each TA muscle with 70 μL of AAV (~1.75 × 10^11^ vg/70 μL). In figure captions, *n* corresponds to the number of muscles analyzed in two or more independent experiments.

### 2.3. Removal and Fixation of TA Muscles 

TA muscles were removed 4 weeks after injection, following euthanasia of mice by cervical dislocation. To perform immunohistochemical analyses, muscles were mounted in tragacanth gum (Fisher Scientific, Hampton, NH, USA) and frozen following different protocols. A one-step fixation protocol consisted of plunging the muscle for at least 1 min in isopentane precooled in liquid nitrogen. Alternatively, a three-step paraformaldehyde (PFA)-sucrose-isopentane protocol consisted of fixing the muscle with 4% paraformaldehyde in PBS for 1 h, followed by immersing it in three successive baths of gradually increasing concentrations of sucrose dissolved in PBS (5% sucrose for 2 h, 10% sucrose for 3 h, and 25% sucrose overnight) at 4 °C. Tissues were finally fixed in isopentane as described above. In parallel, to prepare protein or RNA extracts, muscles were transferred to microcentrifuge tubes and snap-frozen in liquid nitrogen.

### 2.4. Analysis of TA Muscle Protein Extracts

Tissue from half of a snap-frozen TA muscle was resuspended in 145 μL of Laemmli sample buffer (without reducing agent) before heating at 95 °C for 10 min. Samples were homogenized with a Polytron homogenizer (Kinematica, Lucerne, Switzerland) for 15 s and finally centrifuged at 900× *g* (3100 rpm) for 3 min at 4 °C. Protein concentration in the resulting supernatant was measured using the Bradford assay. Finally, Dithiothreitol (DTT) (1 mM final) was added to the supernatant before loading 15 μg of total extracted proteins per well onto 10% polyacrylamide gels. Transfer of proteins from the gel to nitrocellulose membranes, incubation with antibodies, and visualization with enhanced chemiluminescence were done as previously described [11].

### 2.5. Histology of TA Muscle Sections

Sections were cut to 6-μm thickness using a Leica Biosystems CM1950 cryostat (Buffalo Grove, IL, USA), recovered on Superfrost plus glass microscope slides (Thermo Fisher Scientific, Waltham, MA, USA), and stored at −80 °C. 

Muscle sections were incubated at room temperature for 20 min before staining with hematoxylin and eosin, SDH, or Sirius Red. Images were taken with a Leica DM IL inverted microscope equipped with a Qicam digital camera (Qimaging, Surrey, BC, Canada) at the Unité de Biologie Fonctionnelle et Adaptative (BFA) imaging facility. 

For hematoxylin and eosin staining, transverse muscle sections were incubated with 0.7% Harris modified hematoxylin solution (Sigma-Aldrich, St. Louis, MO, USA) for 6 min at room temperature, washed five times with tap water, and stained with 0.5% eosin in acidified 90% ethanol (Sigma-Aldrich) for 1 min at room temperature. Muscle sections were then dehydrated in seven successive baths of gradually increasing concentrations of ethanol solutions (30%, 50%, 70%, 85%, 95%, and 100%, repeated twice) for 2 min each, followed by two baths in Histo-Clear clearing solution (National Diagnostics, Atlanta, GA, USA) for 10 min and 15 min. Tissues were rapidly mounted in VectaMount medium (Vector Laboratories Inc., Burlingame, CA, USA).

For SDH staining, transverse muscle sections were stained by immersing in buffer SDH (0.2 M phosphate buffer containing 5.4% sodium succinate and 0.1% Nitro blue tetrazolium) for 2 h at 37 °C. Stained tissues were washed in water and either mounted directly in Mowiol medium or processed for further immunofluorescence. 

For Sirius Red staining, transverse muscle sections directly frozen in isopentane were post-fixed with 4% formaldehyde in PBS for 2 min, washed in water, and incubated in 100% ethanol for 5 min. Tissues were dried at room temperature (>20 min), and then were incubated in 0.3% Sirius Red in picric acid for 1 h and washed with distilled water. Staining was fixed with two baths of 0.5% acetic acid for 5 min each, dehydrated with three successive baths of 100% ethanol, and cleared with two baths of xylene. Slides were mounted with Eukitt medium (Sigma-Aldrich).

### 2.6. Primary Antibodies

Primary antibodies used included: rabbit anti-FLAG (Sigma-Aldrich); mouse (IgG1) anti-MHC I (clone WB-MHC; Novocastra Laboratories, Leica Microsystemes SAS, Nanterre, France); mouse (IgM) anti-MHC IIX [clone 6H1; Developmental Studies Hybridoma Bank (DSHB), Iowa City, IA, USA]; mouse (IgM) anti-MHC IIB (clone BF-F3; DSHB); mouse (IgG1) anti-MHC IIA (clone SC-71; DSHB); rat (monoclonal) anti-perlecan (Millipore, Billerica, MA, USA); mouse anti-desmin (clone D33; Dako, Agilent, Santa Clara, CA, USA); and rabbit anti-lamins A and C (lamins A/C) [12].

### 2.7. Immunofluorescence of TA Muscle Sections

To assess the integrity of nuclei and the precise subnuclear localization of FLAG-tagged lamins, muscle sections were fixed following the three-step PFA-sucrose-isopentane protocol. For double or triple staining to detect MHC isoforms, muscles were fixed following the one-step isopentane protocol and sections were processed for immunofluorescence either directly (MHC IIB staining) or following a post-fixation step with 2% PFA for 5 min (MHC I, MHC IIX, and MHC IIA staining). 

In all cases, sections were incubated with blocking solution containing 2% goat serum and 2% BSA for 1 h at room temperature and then incubated for 30 min with goat (IgG Fab fragment) anti-mouse IgG (1:100, Jackson ImmunoResearch Laboratories, West Grove, Pennsylvania, United States). Following a PBS wash, sections were incubated with following antibodies:

- Single immunostaining for FLAG-lamin A: Rabbit anti-FLAG (1:350) for 1.5 h at room temperature, followed by donkey anti-rabbit IgG coupled to Cy5 (1:150) for 1 h at room temperature.
- Double immunostaining for desmin/FLAG, which required two successive incubations: (1) mix of mouse anti-desmin (1:50) and rabbit anti-FLAG (1:350) for 1.5 h at room temperature; and (2) mix of goat anti-mouse IgG coupled to fluorescein (488) (1:400) with donkey anti-rabbit IgG coupled to Cy5 (1:150) for 1 h at room temperature.
- Double immunostaining for MHC I/FLAG, which required four successive incubations: (1) mouse anti-MHC 1 (1:20) overnight at 4 °C; (2) goat anti-mouse IgG coupled to fluorescein (488) (1:400) for 1 h at room temperature; (3) rabbit anti-FLAG (1:350) for 1.5 h at room temperature; and (4) donkey anti-rabbit IgG coupled to Cy5 (1:150) for 1 h at room temperature.
- Triple immunostaining for MHC IIB or MHC IIX/FLAG/perlecan, which required six successive incubations: (1) rabbit anti-FLAG (1:350) for 1.5 h at room temperature; (2) donkey anti-rabbit IgG coupled to Cy5 (1:150) for 1 h at room temperature; (3) mouse anti-MHC IIX (1:30) or mouse anti-MHC IIB (1:2) overnight at 4 °C; (4) rabbit anti-mouse IgM coupled to Cy3 (1:250) for 1 h at room temperature; (5) rat anti-perlecan (1:300) for 1.5 h at room temperature; and (6) goat anti-rat coupled to fluorescein (488) (1:400) for 1 h at room temperature.
- Triple immunostaining for MHC IIA/FLAG/perlecan, which required four successive incubations: (1) mouse anti-MHC IIA (1:20) overnight at 4 °C; (2) goat anti-mouse IgG1 coupled to Cy3 (1:250) for 1 h at room temperature; (3) mix of rabbit anti-FLAG (1:350) and rat anti-perlecan (1:300) for 1.5 h at room temperature; and (4) mix of donkey anti-rabbit IgG coupled to Cy5 (1:150) and goat anti-rat coupled to fluorescein (488) (1:400) for 1 h at room temperature.

In all cases, sections were washed in PBS between antibody incubations. Combined detection of SDH, MHCs, and perlecan was done by first processing muscle sections to reveal SDH activity and then marking proteins of interest (MHC isoforms and perlecan), as described above. After incubation, slides were washed in PBS before mounting with Vectashield (or Mowiol) with 4′,6-diamidino-2-phenylindole (DAPI) (Vector Laboratories Inc.). Images were captured using a LSM 700 motorized confocal laser scanning microscope (Zeiss, Oberkochen, Germany) at the BFA imaging facility.

### 2.8. Analysis of TA Muscle Characteristics

TA section size, amount of fibers, and fiber diameter (minimal Feret values) were estimated on whole muscle transverse sections double-stained for FLAG and perlecan. The percentage of fibers expressing the various MHC isoforms was estimated on whole muscle transverse sections triple-stained for MHC (either I or IIA or IIX or IIB), FLAG and perlecan. Images were captured with a confocal laser scanning microscope and analyzed with ImageJ software (ImageJ 1.46r, Wayne Rasband, National Institutes of Health, Bethesda, MD, USA) and a homemade macro [5]. In particular, the percentage of transduction was roughly estimated by measuring the surface of transverse muscle sections that was positively stained for FLAG versus the total section surface area.

### 2.9. Quantitative RT-PCR 

RNA was extracted from TA muscle with Trizol (ThermoFisher Scientific) following manufacturer’s instructions. RNA was used to synthesize cDNA using avian myeloblastosis virus reverse transcriptase with a first strand cDNA synthesis kit with oligo-dT primers (Roche, Basel, Switzerland). Table 1 lists the primers used to amplify each gene. Quantitative RT-PCR was performed in duplicate wells using SYBR green I master mix (Roche) with a Lightcycler 480 II (Roche). Relative gene expression was first estimated using reference gene 18S rRNA and subsequently by normalizing values to target genes expressed in wildtype (WT) samples.

### 2.10. Statistical Analysis

Quantitative results are expressed as means ± standard error of the mean (s.e.m.). Comparisons between samples were performed with the Mann–Whitney test.

## 3. Results

### 3.1. Similar Morphological and Histological Properties of TA Transduced with AAV-FLAG-LA WT or Mutant R388P

**Figure 1:**
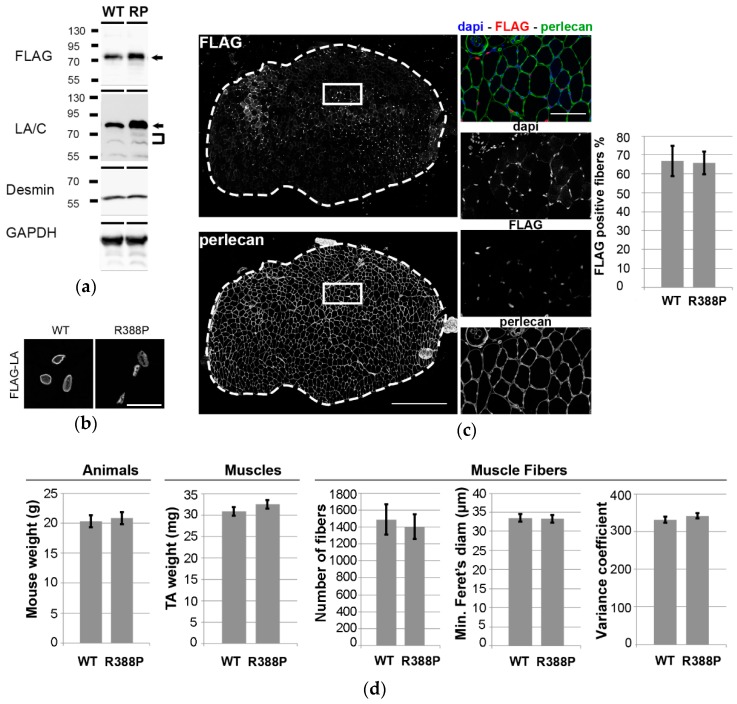
Impact of Adeno-associated virus (AAV)-mediated lamin A (LA) expression on phenotypes of mouse Tibialis anterior (TA) muscles. (**a**) Western blot analysis of whole protein extracts of TA transduced with AAV-LA wild-type (WT) or mutant R388P (RP) tagged with FLAG. Ectopic lamin A (arrows) was detected using either anti-FLAG or anti-lamin A/C antibodies. Endogenous lamin A/C (brackets) was detected using anti-lamin A/C antibodies. Desmin and GAPDH were used as loading controls. (**b**) In situ detection of ectopic lamin A using anti-FLAG antibodies on transverse sections of TA expressing WT or mutant lamin A. Scale bar represents 20 μm. (**c**) Representative images show in situ detection of ectopic lamins, perlecan, and DNA, in a TA muscle section upon expression of WT lamin A. Scale bar represents 500 μm. Areas indicated by white squares are magnified in right panels, where scale bar represents 100 μm. Graph shows the mean percentage of FLAG-positive areas detected on transverse sections (*n* = 8–9). (**d**) Quantification of mouse body weight, TA muscle weight, mean number of fibers, mean minimal Feret’s diameter, and mean variance coefficient of myofiber diameter in transverse TA muscle sections upon expression of WT or mutant lamin A (*n* = 10–11).

Expression of ectopic lamins was assessed one month after injection of AAV into mouse TA muscles. Western blot analyses using anti-FLAG or anti-lamin A/C antibodies detected both ectopic WT and mutant lamin A in muscle protein extracts (Figure 1a). Higher detection of mutant lamin A versus ectopic WT lamin A likely resulted from higher solubilization properties [11].

As expected, immunohistochemistry revealed WT lamin A with dual intranuclear locations, i.e., beneath the nuclear envelope and in the nucleoplasm (Figure 1b). In contrast, mutant lamin A diffused throughout the nucleoplasm, with the exception of nucleolar regions. Accordingly, this unusual lamin A distribution was previously observed in C2C12 myoblasts overexpressing the R388P mutant lamin A [11]. TA transverse muscle sections were stained for FLAG and perlecan, an extracellular matrix proteoglycan, to visualize the borders of each muscle fiber. We observed similarly high transduction efficiency (~67% of muscle fibers) with WT or mutant lamin A (Figure 1c).

Mice expressing WT or mutant lamin A had similar body weights (20 ± 0.5 g and 21 ± 0.5 g, respectively) and TA muscle weights (31 ± 1 mg and 33 ± 1 mg, respectively). To determine more specifically the characteristics of TA expressing WT or mutant lamin A, muscle sections were stained for FLAG and perlecan as above. Sections of muscles expressing WT or mutant lamin A had similar number of fibers (1490 ± 180 and 1405 ± 146, respectively), fiber diameters (34 ± 1 μm and 33 ± 1 μm, respectively), and variance coefficients of fiber diameter (331 and 342, respectively) (Figure 1d).

**Figure 2:**
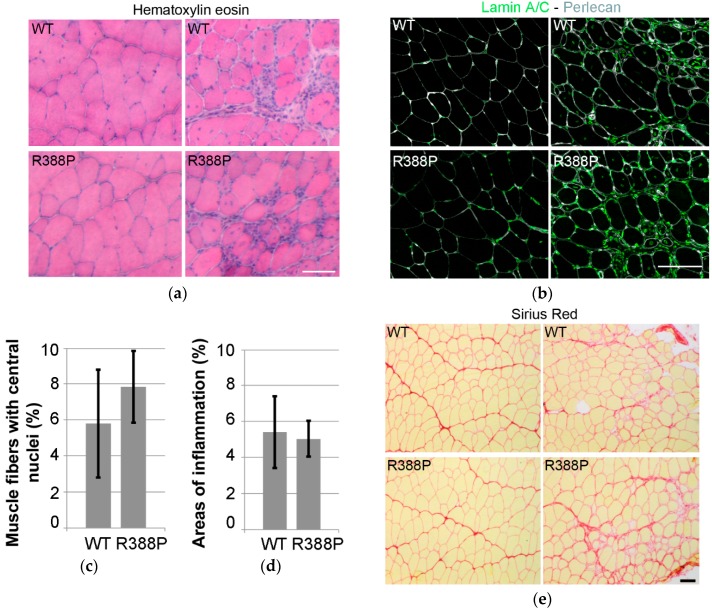
Inflammation, regeneration, and fibrosis in mouse TA muscles expressing AAV-mediated lamin A. (**a**) examples of hematoxylin and eosin staining of transverse muscle sections expressing WT (upper) or R388P mutant (lower) lamin A. (**b**) in situ detection of A-type lamins (ectopic and endogenous; green) and perlecan (grey) on transverse muscle sections expressing WT or R388P lamin A. (**c**) mean percentage ± s.e.m. of myofibers with central nuclei (*n* = 6–7). (**d**) mean percentage ± s.e.m of muscle section areas showing patterns of inflammation (*n* = 7–8). (**e**) examples of Sirius Red staining of transverse muscle sections expressing WT (upper) or mutant (lower) lamin A. Scale bars represent 100 μm.

Eventual defects in TA-transduced muscles were assessed through areas of inflammation or regeneration as indicated by central position of nuclei within fibers. In muscles expressing WT or mutant lamin A, hematoxylin and eosin staining and immunodetection of lamin A/C revealed similar levels of inflammation (5%) (Figure 2a,d) and percentages of muscle fibers with central nuclei (5%–7%) (Figure 2b,c). In addition, Sirius Red staining showed no particular signs of fibrosis in muscle sections expressing either WT or mutant lamin A (Figure 2e).

### 3.2. Expression of Mutant Lamin A Lowers the Frequency of Fast Oxidative Fibers in TA Muscle

**Figure 3:**
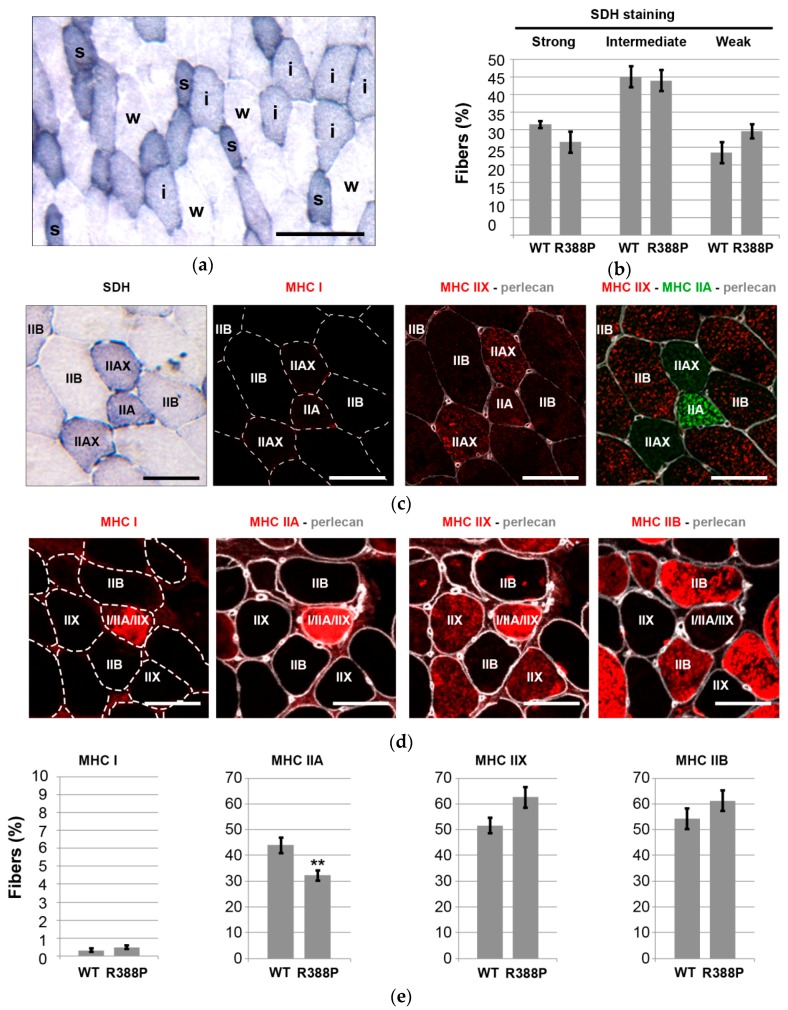
Fiber type distribution analyses in mouse TA muscles expressing AAV-mediated lamin A. (**a**) Representative image show the staining for succinate dehydrogenase (SDH) activity in a transverse sections of TA muscle upon expression of mutant lamin A. Labels of s, i, and w indicating strong, intermediate, and weak SDH staining, respectively. Scale bar represents 100 μm. (**b**) Mean percentage ± s.e.m of fibers with SDH activity relative to total number of fibers in a muscle section expressing WT or R388P mutant lamin A (*n* = 8–11 muscles). (**c**,**d**) Representative images show the staining in successive transverse sections of TA muscle upon expression of mutant lamin A for SDH and Myosin heavy chain (MHC) isoforms MHC I, MHC IIA, MHC IIX, or MHC IIB. Perlecan staining delimited individual fibers. Scale bars represent 50 μm. (**e**) Mean percentage ± s.e.m of fibers expressing MHC I, MHC IIA, MHC IIX, or MHC IIB relative to total number of fibers in a muscle section expressing WT or R388P mutant lamin A (*n* = 8–11 muscles). ** *p* < 0.05 (Mann–Whitney test).

We analyzed SDH mitochondrial enzyme activity on TA transverse muscle sections to determine whether expression of mutant lamin A impacted oxidative properties of muscle fibers. We distinguished three categories of SDH activity—strong, intermediate, or weak—that should correspond to highly oxidative, intermediately oxidative, or non-oxidative fibers, respectively (Figure 3a).

Frequency of non-oxidative fibers (weak SDH staining) was slightly higher in muscles expressing mutant lamin A compared to WT lamin A (Figure 3b), and these were glycolytic fibers that express MHC IIB (Figure 3c). The proportion of two types of oxidative fibers (intermediate or strong SDH staining) appeared slightly lower in muscles expressing mutant lamin A. However, none of these differences were statistically significant. 

Nonetheless, MHC isoform immunostaining (Figure 3c,d) revealed a significantly lower percentage of MHC IIA-positive fibers in TA expressing mutant lamin A (32% ± 2% of fibers) compared to WT lamin A (44% ± 3% of fibers) (*p* < 0.05; Figure 3e). Of note, this category of fibers includes fast oxidative fibers enriched exclusively in MHC IIA as well as hybrid fibers that express both MHC IIA and MHC IIX (Figure 3c). As expected, TA muscle contains a very minor subpopulation of slow oxidative fibers that express MHC I with a similar frequency upon overexpression of either WT or mutant lamin A (0.3%–0.5% of fibers). Percentages of fast glycolytic fibers positive for either MHC IIX or MHC IIB showed a tendency to be slightly higher in TA expressing mutant lamin A in comparison to WT lamin A, although differences were not statistically significant (Figure 3e).

Altogether, SDH and MHC isoform detection pointed to a lower ratio of fast oxidative/fast glycolytic muscle fibers in TA overexpressing mutant lamin A compared to WT lamin A. 

### 3.3. Expression of Mutant Lamin A Alters Expression of Genes Involved in Fiber Type Determination

To decipher underlying mechanisms related to distinct fiber type patterning with expression of WT or mutant lamin A, we analyzed expression of key molecules responsible for fiber type specificity. Expression of different MHC isoforms is partly regulated at the transcriptional level by a set of transcription factors, including MEF2C, myogenin, MyoD and SIX1-Eya1 [7,10]. Thus, we monitored expression of genes encoding such key molecules in AAV-transduced TA muscles using qRT-PCR. 

**Figure 4:**
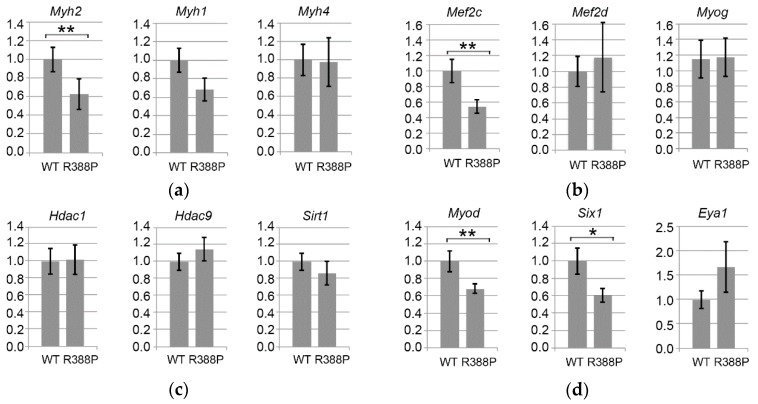

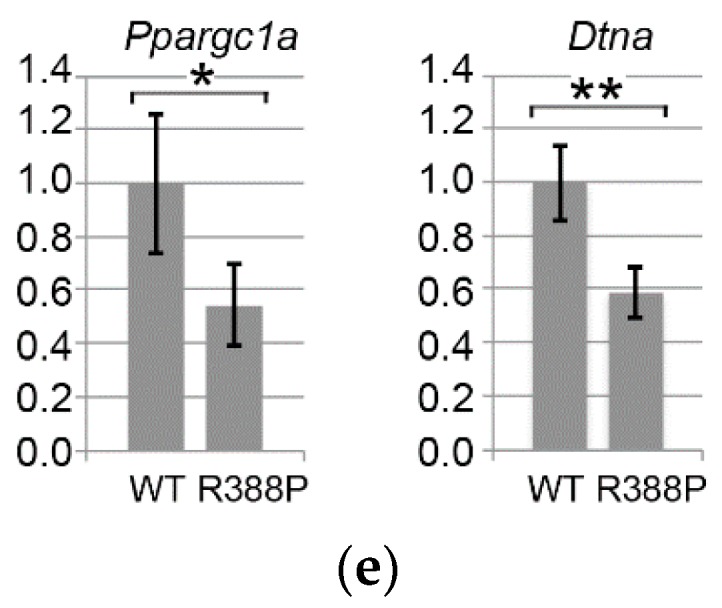
Gene expression related to fiber type determination in TA expressing WT or R388P mutant lamin A. Graphs depict mRNA levels normalized to 18S rRNA for (**a**) *Myh2*, *Myh1*, and *Myh4* mRNAs encoding myosin heavy chains isoforms MHC IIA, MHC IIX, and MHC IIB, respectively; (**b**) *Mef2c*, *Mef2d*, and *Myog* mRNAs encoding key muscle transcription factors MEF2C, MEF2d and Myogenin, respectively; (**c**) *Hdac1*, *Hdac9*, and *Sirt1* mRNAs encoding histone deacetylases HDAC 1, HDAC 9 and SIRT1, respectively; (**d**) *MyoD*, *Six1*, and *Eya1* mRNAs encoding Myod and related co-factors SIX1 and Eya1, respectively; and (**e**) *Ppargc1a* and *Dtna* mRNAs encoding two proteins related to muscular atrophy and dystrophy, PGC1α and Dystrobrevin α, respectively. Data represent mean ± s.e.m (*n* = 12 for WT lamin A; *n* = 10 for mutant lamin A). * *p* = 0.05; ** *p* < 0.05.

We found two-fold significantly lower expression of *Myh2* mRNA (encoding MHC IIA) in muscles expressing mutant lamin A compared to WT lamin A (*p* = 0.02), which fits with the decreased proportion of MHC IIA-positive fibers observed upon expression of mutant lamin A (Figure 4a). In comparison, levels of *Myh1* and *Myh4* mRNA (encoding MHC IIX and MHC IIB, respectively) were not significantly different between muscles expressing WT or mutant lamin A. This suggests that mutant lamin A causes primarily a defect in intermediate gene induction (*Myh2*) to lower the proportion of MHC IIA type fibers, whereas fast gene expression (*Myh4*) may be relatively unaffected.

Among the four members of the MEF2 transcription factor family [13], MEF2C is involved in regulating the proportion of different types of fast fibers (IIA/IIX/IIB) [14]. Downstream of MEF2C is myogenin, a transcription factor belonging to the basic-helix-loop-helix (b-HLH) family that directly regulates *Myh2*. Although myogenin is prevalent in slow muscles, it also is present in fast oxidative fibers of fast TA muscles [15]. Here, we observed significantly reduced expression of *Mef2c* (*p* < 0.05) but similar expression of *Myog* (*p* = 0.97) in TA muscles expressing mutant versus WT lamin A (Figure 4b). Downregulation of *Mef2c* with expression of mutant lamin A does not concern all members of the MEF2 family, however, as *Mef2d* had similar expression in the context of mutant or WT lamin A. 

Upstream of *Mef2c* and *Myog* signaling pathways, there are different classes of acetylase (HAT)/decetylase (HDAC) enzymes that regulate expression of mRNAs and protein activity. In particular, HDACs 5 and 9 in association with HDACs 1 and 3 negatively regulate expression of oxidative fibers by downregulating expression of *Myog* (myogenin) and consequently *Myh2* (MHC IIA) [14,15,16]. We found no change in *Hdac1* or *Hdac9* in TA expressing mutant or WT lamin A (Figure 4c). Thus, lower expression of *Myh2* observed upon expression of mutant lamin A did not correlate with increased expression of *Hdac9*. 

We also measured expression of NAD+-dependent deacetylase SIRT1, which is implicated in deacetylation of MEF2 to inhibit its activity [17]. *Sirt1* expression was similar with WT or mutant lamin A, suggesting that inhibition of MEF2 might occur primarily at the transcriptional level. From these data, we conclude that the distinct MHC IIA fiber type proportion observed with WT or mutant lamin A depends on a change in *Mef2c* expression but independently of a change in *Sirt1* and the *Hdac9-Myog-Myh2* signaling pathway. 

Transcription factor MyoD is another master regulator of skeletal myogenesis through a complex role in muscle fiber identity. Although prevalent in fast muscles, MyoD regulates both fast and slow muscle gene activation, depending on the action of diverse cofactors. For instance, MyoD activates the fast muscle program via SIX1-EYA1 [18]. In accordance with a previous report that MyoD is particularly efficient at *Six1* activation [19], we found significantly lower expression of *Myod* (*p* < 0.05) and *Six1* (*p* = 0.05) upon expression of mutant lamin A compared to WT lamin A (Figure 4d). 

From this observation, one would expect decreased expression of fast genes, specifically with mutant lamin A [19]. However, we measured similar expression of *Myh1* (MHC IIX) and *Myh4* (MHC IIB) in the two lamin A contexts, suggesting that the *Myod-Six1/Eya1* pathway is not sufficiently disturbed with mutant lamin A to influence expression of these fast muscle genes. 

In addition, MyoD can also activate slow muscle gene expression as *Myh7* and *Ppargc1a* in the presence of NFATC1 or SIRT1-PGC1α, respectively. PGC1α is a key molecule involved in different functions, including stimulation of mitochondria biogenesis, which favors oxidative metabolism [18]. In addition, PGC1α triggers expression of genes related to slow fiber types more specifically in slow/oxidative muscles, notably by activating PPARβ/δ. Indeed, we found significantly lower expression of *Myod* (Figure 4d) and *Ppargc1a* (*p* = 0.05; Figure 4e) mRNAs specifically with mutant lamin A. These results suggest that the decreased proportion of fast oxidative fibers in TA expressing mutant lamin A involves deregulation of the PGC1α pathway.

### 3.4. Expression of Dystrobrevin α in TA Muscles Expressing WT or Mutant Lamin A 

Dystrobrevin-α, encoded by *Dtna*, belongs to a family of dystrophin-related proteins that, together with dystrophin, syntrophin, α-dystroglycan (α-DG), β-dystroglycan (β-DG), sarcoglycan complex (SGC), and sarcospan (SPN), form a scaffold for signaling molecules located at the sarcolemma [20]. Expression of dystrobrevin-α is related to muscle diseases (see discussion). In addition, ChIPseq analysis in HeLa cells shows that WT and mutant (R388P) lamin A differentially bind *Dtna* [21]. We observed a 2.2-fold reduction in *Dtna* expression (*p* < 0.01) in mouse TA expressing mutant lamin A compared to WT lamin A (Figure 4e). These results suggest that deregulation of *Dtna* expression may induce some muscle alterations specifically in the context of mutant lamin A.

## 4. Discussion

### 4.1. Validation of the AAV Experimental Model to Explore Laminopathies

A pediatric patient bearing a heterozygous p.R388P *LMNA* mutation presented early difficulties climbing stairs and walking distances and an inability to run. Examination of the patient’s muscle biopsy showed dystrophic changes [11]. However, we did not detect dystrophic characteristics in TA muscle sections of mice locally expressing mutant lamin A due to AAV injection. However, the literature reports that only more severe forms of myopathies, such as L-CMD, show prominent dystrophic and/or inflammatory changes. Moreover, congenital FTD might be a milder form of early-onset LMNA myopathy [8].

Here, it seems that mice injected with AAV encoding mutant lamin A develop a milder form of muscle alteration than the original human patient with L-CMD. There are multiple possible reasons for this difference. Of note, our experiments analyzed muscles only one month after injection of AAV encoding ectopic lamin A in adult mice. Whether more dramatic effects on muscle morphology might be induced in younger mice or at other timepoints after AAV injection remain unknown. Although we chose to inject TA muscles with AAV for practical reasons, it is also possible that injection of other types of muscles—such as soleus, which is a slow muscle—might trigger more dramatic muscle changes. Moreover, particularly in the context of laminopathies, mice often require homozygous mutation to present similar phenotypes to those induced by heterozygous mutation in humans [4]. Nevertheless, injection of AAV encoding lamins in mice resembles more closely a heterozygous genotype. Taking into account these limits of our experimental approach, our data nonetheless validate the injection of AAV encoding lamins in mouse muscles to explore the impact of lamin A on muscle integrity.

### 4.2. Role of Key Transcription Factors in Laminopathy Induced by R388P Mutant Lamin A

**Figure 5:**
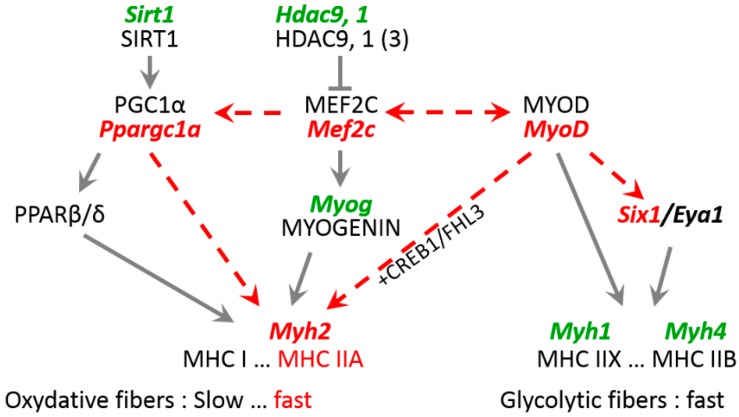
Expression pathways for genes encoding key factors related to *Myh2* and MHC IIA expression that are induced by expression of mutant lamin A in mouse TA. Bold, italic, green text indicates no change in mRNA expression; bold, italic, red text indicates reduced mRNA expression. Dashed lines indicate hypothetical altered pathways that decrease levels of *Myh2* mRNA and MHC IIA.

Our results indicate that expression of mutant lamin A significantly reduced *Myh2* expression, in accordance with with lower in situ detection of MHC IIA. While MyoD directly regulates *Mef2c* mRNA expression, MEF2C regulates *Myh2* mRNA expression indirectly via its capacity to regulate *Myog* mRNA expression (Figure 5). However, despite the decreased *Mef2c* mRNA expression, absence of altered *Myog* mRNA expression in the context of mutant lamin A suggests that this pathway (MyoD-MEF2C-myogenin) is not responsible for lower *Myh2* mRNA and MHC IIA expression. Instead, MyoD can also activate *Myh2* transcription directly in the presence of the CREB1-FHL3 complex [10]. Therefore, assuming that lower levels of *Myod* mRNA correspond to lower levels of MyoD, we propose that reduced *MyoD* directly leads to less efficient expression of *Myh2*. 

Moreover, we propose that, in the context of mutant lamin A, MEF2C might also impact the fast oxidative pattern of fibers. This may happen not through regulation of myosin heavy chain genes, but through downregulation of *Ppargc1a*, which encodes a transcriptional coactivator that induces key mitochondrial genes. Indeed, lower *Mef2c* levels correlate with the lower expression of *Ppargc1a* and reduced ratio of fast oxidative/fast glycolytic muscle fibers in TA expressing mutant lamin A (Figure 5).

In addition, mutant lamin A also alters regulation of *Dtna*, which encodes one of the protein partners constituting the dystrophin-glycoprotean complex (DGC) at the sarcolemma. In humans, single nucleotide mutations in *Dtna* are related to pathologies, including left ventricular non-compaction with heart congenital disease [22] and Meniere’s disease [23]. In mice, dystrobrevin-α knockout leads to a mild form of dystrophy associated with impaired DGC-dependent signaling, but no disruption of DGC at the sarcolemma [24]. One can thus expect that lower *Dtna* expression would lead to reduced expression of dystrobrevin-α and consequently affect DGC-dependent signaling, including those related to calmodulin kinase II, focal adhesion kinase, mitogen-activated protein kinase kinase 2, neuronal nitric oxide synthase PIP2, and phosphatidylinositol 4,5-bisphosphate [25].

Taking into account the diversity of signaling pathways (MEF2C, MyoD, PGC1α, and dystrobrevin-α) altered in response to mutant lamin A, one might expect muscle alterations at different levels, including fiber type specification, muscle fiber contraction, and muscle endurance capacity.

### 4.3. Mechanisms Underlying Deregulation of Key Genes upon Expression of R388P Mutant Lamin A

In nuclei, A-type lamins are mainly detected as a major pool underlying the nuclear envelope and another pool distributed throughout the nucleoplasm. By which mechanism could mutant lamin A alter expression of a subset of genes including *Myh2*, *MyoD* and *Dtna*? A-type lamins contribute to genome organization and modulation of local gene expression [26,27]. Moreover, the two pools of WT A-type lamins likely bind distinct chromatin types, more condensed and gene-poor at the nuclear periphery, but more open and gene-rich within the nucleoplasm [28]. In cells, mutant and WT lamin A have strikingly different behaviors, as illustrated by depletion from the nuclear envelope but accumulation within the nucleoplasm and increased solubilization properties of mutant lamin A [11]. Moreover, chromatin immunoprecipitation studies revealed striking differences in the nature of chromatin bound by mutant versus WT lamin A [21]. Thus, one can expect that mutant lamin A would modulate expression of a subset of genes differently than WT lamin A. 

Our current working hypothesis is that mutant lamin A, by binding to distinct genome regions, alters expression of numerous genes, including *Myh2*, *MyoD*, and *Dtna*. Alternatively, it is possible that the distinct gene transcription profile observed in TA expressing mutant lamin A involves changes in the capacity of this mutant lamin A to interact with some of its partners [29] known to regulate gene expression, including HDACs (as SIRT1) [30] and histone methyltransferases (as suv39h1) [31].

## 5. Conclusions

In conclusion, the major change we observed in a TA transiently expressing mutant compared to WT lamin A was a decreased amount of fast oxidative muscle fibers, which express MHC IIA. Moreover, gene expression analysis suggested altered MyoD-MEF2C expression/function at the core of the TA fiber type alteration. At last, we propose from our data that viral-mediated expression of lamin A in mouse skeletal muscles can be considered as an alternative pertinent model to reveal specific muscle alterations due to lamin A mutants. 

## Figures and Tables

**Table 1:** Primers used for qRT-PCR.

Gene	Forward Primer (5′… 3′)	Reverse Primer (5′… 3′)
*18S rRNA*	*CGGCTACCACATCCAAGGAA*	*TATACGCTATTGGAGCTGGAA*
*Myh2*	*AAAGCTCCAAGGACCCTCTT*	*AGCTCATGACTGCTGAACTC*
*Myh1*	*CCAAGGAGGAGGAACAGCAG*	*TTTCGTCTAGCTGGCGTGAG*
*Myh4*	*GTCCTTCCTCAAACCCTTAAAGT*	*ATCTCAGCGTCGGAACTCAT*
*Mef2c*	*AGAGTTTGGACAACAAAGCCC*	*CACGCTTCACTTCATCTCTCC*
*Mef2d*	*AGGGAGGCAAAGGGTTAATGC*	*CCTGGCTGAGTAAACTTGGTG*
*Myog*	*GTCCCAACCCAGGAGATCAT*	*CTGTCCACGATGGACGTAAG*
*Myod*	*CTGCTCTGATGGCATGATGG*	*TGTAGTAGGCGGTGTCGTAG*
*Six1*	*AAGAACGAGAGCGTGCTCAA*	*CATCCCTTCAAGGCCCCAAT*
*Eya1*	*TGGCCCTACCCCTTCCCCAC*	*TGACAATCCACTTTCCGTCTT*
*Hdac1*	*TACTACGACGGGGATGTTGGAAAC*	*TCCTCAGCATTGGCTTTGTGA*
*Hdac9*	*GCGGTCCAGGTTAAAACAGA*	*GAGCTGAAGCCTCATTTTCG*
*Sirt1*	*AGAACCACCAAAGCGGAAA*	*TCCCACAGGAGACAGAAACC*
*Ppargc1a*	*CGGAAATCATATCCAACCAG*	*TGAGGACCGCTAGCAAGTTTG*
*Dtna*	*GCAGAGATGAGGGCTCAAG*	*TGACGTTCCAAATGTCCACC*
